# Supplementary material for: Toxicological Impacts and Mechanistic Insights of Bisphenol a on Clear Cell Renal Cell Carcinoma Progression: A Network Toxicology, Machine Learning and Molecular Docking Study
Source: Biomedicines. 2025 Nov 13;13(11):2778. doi: 10.3390/biomedicines13112778 (PMC12650149; doi:10.3390/biomedicines13112778)
Supplement: Supplementary file 1 [file biomedicines-13-02778-s001.zip › Supplementary Table S1.pdf]

Supplementary Table S1

Supplementary table S1A. Website URLs for database and tools used in present study.

	Steps	Database/Tools	Website URL
1	Biotoxicity prediction	ProTox-3.0	https://tox.charite.de/protox3/
		ADMETlab 2.0	https://admetmesh.scbdd.com/
		Vnn-ADMET	https://vnnadmet.bhsai.org/vnnadmet/about.xhtml
2	SMILES	PubChem platform	https://pubchem.ncbi.nlm.nih.gov/
3	Drug Target Identification	Swisstargetprediction	http://www.swisstargetprediction.ch/
		CheMBL	https://www.ebi.ac.uk/chembl/
		STITCH	http://stitch.embl.de/
4	Disease Target Identification	GeneCards	http://www.genecards.org/
		TTD	https://db.idrblab.net/ttd/
		OMIM	https://www.omim.org/
5	Drug-Disease Target Overlap Analysis	Venny 2.1	https://bioinfogp.cnb.csic.es/tools/venny/index.html
6	PPI Network	STRING	https://STRINGdb.org/
		Cytoscape software3.91	https://cytoscape.org
7	Enrichment Analysis	DAVID	https://david.ncifcrf.gov/
		FUMA	https://fuma.ctglab.nl/
		WeiShengXin	https://www.bioinformatics.com.cn/
		Metascape	https://metascape.org/gp/index.html#/
8	Prognostic model	The Cancer Genome Atlas (TCGA) database	https://portal.gdc.cancer.gov/
		ArrayExpress	https://www.ebi.ac.uk/biostudies/arrayexpress
		cBioportal	https://www.cbioportal.org/
9	Molecular Docking Simulation	Alphafold	https://alphafold.com/
		Uniprot	https://www.uniprot.org/
		RCSB PDB	https://www.rcsb.org
		CB-DOCK	https://cadd.labshare.cn/cb-dock2/
		Discovery-studio	https://www.3ds.com/products/biovia/discovery-studio

Supplementary table S1B. Potential targets of BPA-induced renal toxicity (114).

AR	MMP9	IDO1	JAK3	CYP2A6	MAP2K2
ESR1	MMP1	DYRK1B	JAK2	CSNK1E	EDNRA
ESR2	MMP2	HDAC6	MAP2K1	MAOA	MC4R
ALOX5	NR1H3	CTSK	REN	ADORA2A	CCN4
CA2	IGF1R	FGFR1	PRKCE	TSHR	HRH2
CA4	BRAF	AKT1	CCNE1	FLT1	NFE2L2
PTGS1	PARP1	GABBR1	PTK2	KRAS	CYP2C9
BCL2L1	UPP1	EPHX2	CYP2E1	CXCR1	CYP2D6
BCL2	ACHE	MYLK	CYP1A2	ADRB1	GSK3B
ALOX15	HSP90AA1	CYP19A1	CLK2	CCR2	LCK
ALOX12	TRAP1	RAF1	RHEB	CCR4	ADAM9
SLC6A2	HSP90AB1	ROCK1	ELANE	FYN	HSPA8
PTGS2	RIPK2	PRKACA	ADRB2	PRKCA	NOS3
SRD5A1	ALPL	KCNN4	ADRA1D	PTPRC	LEPR
SLC6A3	FASN	CDK2	RXRA	CYP3A4	
CHRM3	DHFR	CCNA1	HPGD	CASP1	
PLA2G2A	CDK5	CCNA2	PPARG	MAPK14	
CA14	NR1H4	MAPK10	NR3C1	CCR5	
TYR	HDAC1	MAPK9	HMGCR	CYP2S1	
FTO	NR1I2	PAK1	GALC	GLS	

Supplementary table S1C. The enrichment chart of GO function analysis of potential targets(top10).

Category	GO term	Gene Ratio	Gene count	P-value
BP	protein phosphorylation	21.9298246	25	1.10E-18
	phosphorylation	25.4385965	29	1.51E-17
	positive regulation of MAPK cascade	11.4035088	13	8.40E-10
	long-chain fatty acid biosynthetic process	6.14035088	7	1.56E-09
	peptidyl-tyrosine phosphorylation	7.01754386	8	1.36E-08
	peptidyl-serine phosphorylation	9.64912281	11	2.34E-08
	signal transduction	22.8070175	26	2.61E-08
	monoterpenoid metabolic process	4.38596491	5	3.52E-08
	MAPK cascade	8.77192982	10	4.51E-08
	inflammatory response	10.5263158	12	3.09E-05
CC	plasma membrane	57.0175439	65	1.10E-18
	cytosol	56.1403509	64	1.51E-17
	cytoplasm	52.6315789	60	8.40E-10
	mitochondrion	23.6842105	27	1.56E-09
	receptor complex	9.64912281	11	1.36E-08
	perinuclear region of cytoplasm	15.7894737	18	2.34E-08
	neuronal cell body	11.4035088	13	2.61E-08
	intracellular membrane-bounded organelle	14.9122807	17	3.52E-08
	nucleoplasm	35.9649123	41	4.51E-08
	endoplasmic reticulum membrane	15.7894737	18	3.09E-05
MF	enzyme binding	18.4210526	21	4.64E-14
	protein serine kinase activity	17.5438596	20	3.98E-13
	protein tyrosine kinase activity	11.4035088	13	9.82E-13
	protein kinase activity	14.9122807	17	6.68E-12
	protein serine/threonine kinase activity	16.6666667	19	8.83E-12
	ATP binding	29.8245614	34	2.80E-11
	heme binding	11.4035088	13	1.15E-10
	nuclear receptor activity	7.89473684	9	8.92E-10
	iron ion binding	9.64912281	11	9.58E-09
	identical protein binding	28.0701754	32	1.11E-08

Supplementary table S1D. The enrichment chart of KEGG function analysis of potential targets (Top 20).

KEGG Terms	Pathways	Gene Ratio	Gene Count	FDR	P-Value
hsa05200	Pathways in cancer	32.45614	37	5.03E-16	4.84E-18
hsa01522	Endocrine resistance	15.78947	18	1.22E-13	2.34E-15
hsa05417	Lipid and atherosclerosis	20.17544	23	3.87E-13	1.12E-14
hsa05235	PD-L1 expression and PD-1 checkpoint pathway in cancer	7.017544	8	1.53E-04	1.15E-04
hsa04660	T cell receptor signaling pathway	11.40351	13	1.39E-07	2.80E-08
hsa05207	Chemical carcinogenesis - receptor activation	17.54386	20	1.96E-10	1.13E-11
hsa04662	B cell receptor signaling pathway	5.263158	6	0.005218	0.005218
hsa04915	Estrogen signaling pathway	14.03509	16	1.55E-09	1.20E-10
hsa04917	Prolactin signaling pathway	10.52632	12	9.58E-09	8.29E-10
hsa04659	Th17 cell differentiation	7.894737	9	8.00E-05	5.23E-05
hsa04151	PI3K-Akt signaling pathway	19.29825	22	2.14E-08	2.27E-09
hsa04071	Sphingolipid signaling pathway	12.2807	14	2.29E-08	2.64E-09
hsa01521	EGFR tyrosine kinase inhibitor resistance	10.52632	12	2.41E-08	3.09E-09
hsa05205	Proteoglycans in cancer	14.91228	17	2.41E-08	3.24E-09
hsa04657	IL-17 signaling pathway	7.894737	9	3.83E-05	2.06E-05
hsa04012	ErbB signaling pathway	10.52632	12	4.41E-08	6.79E-09
hsa04664	Fc epsilon RI signaling pathway	9.649123	11	6.06E-08	9.91E-09
hsa04062	Chemokine signaling pathway	14.03509	16	6.88E-08	1.19E-08
hsa04024	cAMP signaling pathway	14.03509	16	4.11E-07	9.88E-08
hsa04010	MAPK signaling pathway	14.03509	16	9.11E-06	3.67E-06

Supplementary table S1E. Correlation coefficient of prognostic model constructed by 23 screened genes.

Gene	Coefficient	Absolute_Effect
CHRM3	-0.177394829	0.177394829
GABBR1	0.171960465	0.171960465
CCR4	-0.148654096	0.148654096
KCNN4	0.12685455	0.12685455
PRKCE	-0.124408593	0.124408593
CYP2C9	0.115484784	0.115484784
HPGD	-0.115404584	0.115404584
FASN	0.113610201	0.113610201
CA14	-0.11020645	0.11020645
CCNA2	0.104944657	0.104944657
AR	-0.097284256	0.097284256
CSNK1E	0.079144372	0.079144372
PLA2G2A	0.078022385	0.078022385
DYRK1B	-0.074395016	0.074395016
RHEB	0.067168437	0.067168437
MMP1	0.066276727	0.066276727
BCL2L1	-0.065983167	0.065983167
IDO1	0.059521112	0.059521112
HDAC1	0.059438695	0.059438695
ADRB2	-0.056058017	0.056058017
CYP2S1	-0.053160362	0.053160362
EPHX2	-0.048790729	0.048790729
CCNE1	0.041180006	0.041180006

Supplementary table S1F. Prognostic signatures of renal cell carcinoma retrieved from independent studies.

Model	PMID	Cancer	Author	Coef	symbol
Lou, 2025	39536695	ccRCC	Luo	-178.065	FGFBP2
Lou, 2025	39536695	ccRCC	Luo	103.1131	IFNG
Lou, 2025	39536695	ccRCC	Luo	258.1198	PLAC8
Lou, 2025	39536695	ccRCC	Luo	21.04592	CD7
Lou, 2025	39536695	ccRCC	Luo	88.32087	ARL4C
Lou, 2025	39536695	ccRCC	Luo	128.5104	EVL
Lou, 2025	39536695	ccRCC	Luo	-81.8749	IL2RG
Lou, 2025	39536695	ccRCC	Luo	-222.672	SPN
Lou, 2025	39536695	ccRCC	Luo	-27.1649	CYBA
Lou, 2025	39536695	ccRCC	Luo	2.810812	APOBEC3G
Lou, 2025	39536695	ccRCC	Luo	433.7718	ARHGAP9
Lou, 2025	39536695	ccRCC	Luo	25.96993	LIMD2
Lou, 2025	39536695	ccRCC	Luo	-345.09	ABI3
Lou, 2025	39536695	ccRCC	Luo	153.7292	METRNL
Lou, 2025	39536695	ccRCC	Luo	207.264	EMP3
Lou, 2025	39536695	ccRCC	Luo	-345.611	RIPOR2
Lou, 2025	39536695	ccRCC	Luo	-171.061	HSPA8
Lou, 2025	39536695	ccRCC	Luo	45.24195	IDI1
Zhao,2021	34178024	ccRCC	Zhao	0.1581	CARS1
Zhao,2021	34178024	ccRCC	Zhao	0.004	CD44
Zhao,2021	34178024	ccRCC	Zhao	0.1968	FANCD2
Zhao,2021	34178024	ccRCC	Zhao	-0.0464	HMGCR
Zhao,2021	34178024	ccRCC	Zhao	-0.0091	NCOA4
Zhao,2021	34178024	ccRCC	Zhao	0.0225	SLC7A11
Zhao,2021	34178024	ccRCC	Zhao	0.0352	ACACA
Li,2023	37554538	ccRCC	Li	0.20534	CDKN1A
Li,2023	37554538	ccRCC	Li	-0.12909	NCOA4
Choudhury,2015	25018036	ccRCC	Choudhury	-0.0182	CXCL5
Choudhury,2015	25018036	ccRCC	Choudhury	-0.0364	EFNA5
Choudhury,2015	25018036	ccRCC	Choudhury	0.0743	EMCN
Choudhury,2015	25018036	ccRCC	Choudhury	-0.059	LAMB3
Choudhury,2015	25018036	ccRCC	Choudhury	0.0506	PLG
Choudhury,2015	25018036	ccRCC	Choudhury	-0.132	PRAME
Choudhury,2015	25018036	ccRCC	Choudhury	-0.055	RARRES1
Choudhury,2015	25018036	ccRCC	Choudhury	0.105	SLC6A19
