# Supplementary figures and images for: Toxicological Impacts and Mechanistic Insights of Bisphenol a on Clear Cell Renal Cell Carcinoma Progression: A Network Toxicology, Machine Learning and Molecular Docking Study
Source: Biomedicines. 2025 Nov 13;13(11):2778. doi: 10.3390/biomedicines13112778 (PMC12650149; doi:10.3390/biomedicines13112778)

Brier score (IPCW, event formulation)

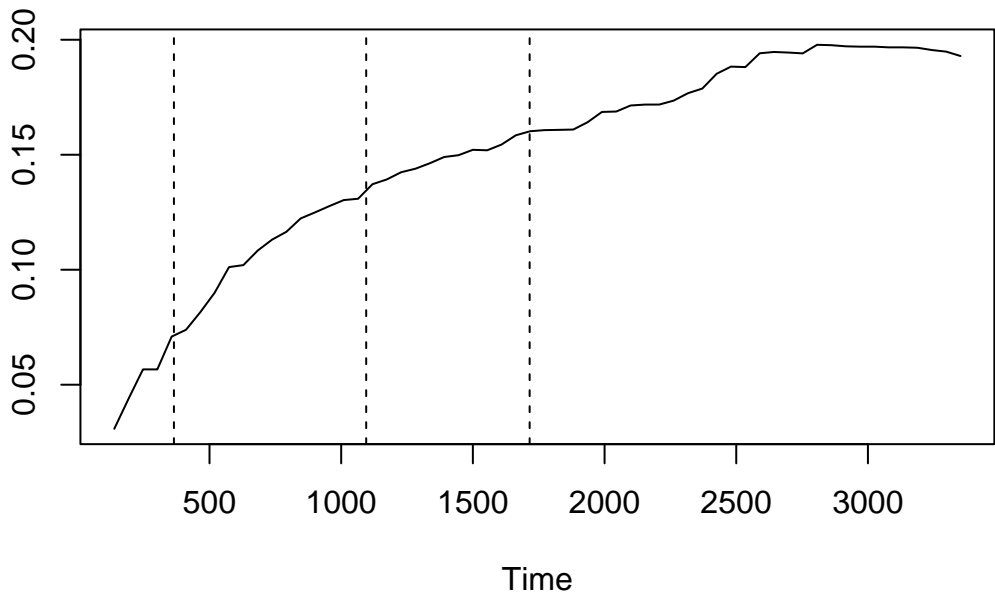

Supplement: Supplementary file 1 [file biomedicines-13-02778-s001.zip › Supplementary Figure S1.pdf]
